# miR-29c-3p represses the angiogenesis of esophageal squamous cell carcinoma by targeting SERPINH1 to regulate the Wnt signaling pathway

**DOI:** 10.1590/acb385223

**Published:** 2023-12-04

**Authors:** Desheng Wei, Zhifeng Ma, Ting Zhu, Haiyong Wang, Bin Wang, Linhai Fu, Guangmao Yu

**Affiliations:** 1 Shaoxing People’s Hospital – Department of Thoracic Surgery – Shaoxing – Zhejiang Province, China.; 2 Zhejiang University – School of Medicine – Shaoxing – Zhejiang Province, China.

**Keywords:** MicroRNAs, HSP47 Heat-Shock Proteins, Esophageal Squamous Cell Carcinoma, Angiogenic Proteins, Wnt Signaling Pathway

## Abstract

**Purpose:**

Esophageal squamous cell carcinoma (ESCC) is characterized by early metastasis and late diagnosis. miR-29c-3p is confirmed to repress angiogenesis in multiple tumor types. Yet, the functions of miR-29c-3p in the mechanism of ESCC angiogenesis, which were not sufficiently explored previously, were exactly what we investigated here at the molecular level.

**Methods:**

The mRNA level of miR-29c-3p and Serpin peptidase inhibitor clade H member 1 (SERPINH1) in ESCC tissues were assessed via bioinformatics analysis. Thereafter, miR-29c-3p and SERPINH1 (HSP47) mRNA level in ESCC cell lines was evaluated via quantitative real-time polymerase chain reaction. The effects of abnormal miR-29c-3p and SERPINH1 expression on ESCC cell viability, proliferation, migration, invasion, and HUVEC angiogenesis were examined via CCK8, colony formation, transwell, and angiogenesis assays, respectively. The protein levels of SERPINH1, vascular endothelial growth factor-A (VEGFA), Wnt-1, ?-catenin, and p-?-catenin were evaluated via Western blot. Expression of VEGFA secreted by ESCC cells was measured via enzyme-linked immunosorbent assay. Treatment with the Wnt activator BML-284 further revealed the way miR-29c-3p mediated the Wnt signaling pathway and its effects on angiogenesis.

**Results:**

Herein, we revealed a decrease of miR-29c-3p expression in ESCC tissues and cells, while the overexpressed miR-29c-3p could remarkably suppress ESCC cell progression, as well as HUVEC angiogenesis. Meanwhile, overexpressed miR-29c-3p notably downregulated VEGFA and repressed the Wnt signaling pathway. Treatment with the Wnt activator BML-284 could reverse the inhibition of HUVEC angiogenesis caused by miR-29c-3p. SERPINH1 was a downstream target of miR-29c-3p. SERPINH1 knockdown suppressed the malignant phenotypes of ESCC cells and impeded the Wnt signaling activation, while such suppression was reversed through miR-29c-3p inhibitor.

**Conclusions:**

We confirmed the mechanism that miR-29c-3p targeted SERPINH1, thus regulating angiogenesis in ESCC through the Wnt signaling pathway. It improves the understanding of angiogenesis in ESCC and offers new ideas for the research of ESCC treatment strategies in the future.

## Introduction

As a main subtype of esophageal cancer, esophageal squamous cell carcinoma (ESCC) covers around 90% of the total cases in China. This disease is estimated to cause more than 375,000 deaths and 477,900 new cases per year, and it is a malignant tumor with high mortality and poor prognosis^1^^,^ 2. Tumor angiogenesis is activated when there are more proangiogenic molecules like vascular endothelial growth factor (VEGF) than antiangiogenic molecules^3^^,^ 4, which is one of the key factors contributing to the ESCC aggressiveness and patients’ poor prognosis^5^. Currently, tumor angiogenesis is an important target for cancer treatment. Therefore, further exploration of the angiogenesis mechanism at the molecular level might provide ESCC patients with a more effective treatment.

As a kind of small non-coding RNAs, microRNAs (miRNAs) function in silencing genes and repressing gene translation through binding with target mRNAs^6^. Recent studies demonstrated that miRNAs were critical to modulating tumor cell malignant progression. For example, in hepatocellular carcinoma, miR-29c-3p suppresses cell migration, proliferation, and tumor growth via DNMT3B and LATS1-related Hippo signaling pathways, suggesting its potential as a therapeutic target^7^, and miR-29c-3p can get recruited by Circ-001971, thereby increasing the VEGFA level to accelerate cell proliferation, invasion, and angiogenesis of colon cancer^8^. In reports on ESCC, miR-29c-3p targets the CCNA2-mediated p53 signaling pathway, represses malignant phenotypes of ESCC cells and arrests cells in G0/G1 phase^9^. It followed from the above that miR-29c-3p is a pivotal modulator for repressing tumor malignant progression. However, whether miR-29c-3p could be involved in regulating angiogenesis in ESCC has not been investigated. The mechanism that miR-29c-3p repressed ESCC progression still needs to be perfected.

Angiogenesis is a complex and dynamical way that is modulated via loads of molecules and signaling pathways^10^. Notably, one publication found that Wnt/β-catenin signaling is pivotal to the angiogenic process^11^. For instance, in lung adenocarcinoma, GOLPH3 facilitates angiogenesis via initiating Wnt/β-catenin signaling pathway^12^. Song et al.^13^ discovered that in colorectal cancer lncRNA GAS5 represses metastasis, invasion, and angiogenesis through suppressing the initiation of the Wnt/β-catenin signaling pathway. The mentioned findings formed a basis on which we conjectured that miR-29c-3p could manipulate ESCC angiogenesis via the Wnt signaling pathway.

Herein, in ESCC, miR-29c-3p was remarkably lowly expressed, and SERPINH1 (HSP47) expression was prominently high. Subsequently, *in-vitro* cell experiments confirmed that miR-29c-3p targeted SERPINH1, thereby repressing angiogenesis and inactivating the Wnt signaling pathway in ESCC cells. We revealed the mechanism by which miR-29c-3p regulated angiogenesis in ESCC at the molecular level, providing strong evidence for the development of miRNA-targeted ESCC regimens.

## Methods

### Bioinformatics analysis

Downloaded from TCGA database, miRNA expression data of ESCC included 13 normal samples and 96 ESCC tissue samples, and mRNA expression data of ESCC included 11 normal samples and 81 ESCC tissue samples. miR-29c-3p and SERPINH1 expression levels in the ESCC dataset were analyzed via the Wilcoxon’s test. TargetScan speculated the downstream binding sites of miRNAs. Pearson’s correlation coefficient was used to analyze the correlation of miR-29c-3p and SERPINH1.

### Cell culture and transfection

Normal human esophageal squamous epithelial cell line Het-1A (BNCC337688), human ESCC cell lines KYSE-150 (BNCC100428), KYSE270 (BNCC340202), KYSE70 (BNCC340532), and human umbilical vein endothelial cell line (HUVEC) (BNCC337632) were procured from BeNa Culture Collection (China). We developed the cell lines into Roswell Park Memorial Institute-1640+10% fetal bovine serum (FBS) medium and kept it in an incubator at 37°C with 5% CO_2_.

miR-29c-3p mimic (miR-mimic), miR-29c-3p inhibitor (miR-inhibitor), sh-SERPINH1 and their matched negative controls (mimic-NC, NC-inhibitor, sh-NC) were synthesized through GenePharma (China). Complying with the instruction, we applied Lipofectamine 2000 (Invitrogen, United States of America) to transfect plasmids. Followed by 24 hours of cultivation, we harvested the cells for subsequent experiments.

### CCK-8 assay

Cell proliferation assay was performed on the cells using CCK-8 (ab228554, Abcam, United Kingdom). We seeded ESCC cells on 96-well plates with each well per 2×10^3^ cells. Cells were cultivated for 0, 24, 48, and 72 hours at 37 °C in a 5% CO_2_ incubator. At each time point, 10 μL of CCK-8 solution was introduced to each well for 2 hours of maintenance. Optical density (at 460 nm) was assessed with a microplate reader, and growth curves were plotted.

### Cell colony formation

Eight hundred cells per well were introduced to six-well plates. The incubation system was kept under 5% CO_2_ at 37°C until colonies were visible. Then, methanol replaced the previous medium for 30 min of cell fixation, and 0.1% crystal violet was introduced for 10 min staining. After PBS-washes and air drying, we observed and calculated the colonies with a microscope. Three plates were detected for each group.

### Transwell method for cell migration and invasion determination

Via transwell chambers (Corning, United States of America), both cell migration and invasion analyses were processed. For invasion assays, we coated the membrane of the upper chamber with Matrigel (BD Biosciences, United States of America), and 2×10^4^ cells/well were introduced to the upper chamber and immersed in a serum-free medium. Dulbecco’s modified eagle medium (DMEM)+10% FBS was introduced to the lower chambers. Cells were maintained under 5% CO_2_ at 37°C for 48 hours. While a cotton swab was used to clear those non-migratory or non-invasive cells on the upper surface of the membrane, the migratory and invasive cells on the other side of the membrane were subjected to fixation with 4% paraformaldehyde and staining with 0.1% crystal violet. The quantity of migrated and invaded cells was counted three times under a microscope. For migration assay, all experimental steps were the same as those of invasion assay, except that no Matrigel was added in the upper chamber.

### Angiogenesis assay

Fifty μL of Matrigel (Corning, United States of America) was introduced to 96-well culture plates followed by 30 min of polymerization at 37°C. HUVEC suspension was moved to wells of 96-well plates per 3×10^4^ cells/well, and ESCC cell conditioned medium (CM) containing sh-SERPINH1, miR-29c-3p mimic, miR-29c-3p inhibitor, and their corresponding negative controls was individually introduced to wells. Six hours after the cultivation, three replicate wells were set for each group with three fields selected in each well.

ESCC cells were treated with 1 μM Wnt activator BML-284 (HY-19987, MedChemExpress, United States of America)^14^. Cell grouping settings were: CM (mimic-NC+DMSO), CM (miR-mimic+DMSO), CM (mimic-NC+BML-284), and CM (miR-mimic+BML-284). The remaining steps were the same as what we already mentioned.

### Quantitative real-time polymerase chain reaction

The specific steps of quantitative real-time polymerase chain reaction (qRT-PCR) analysis were described previously^15^. The brief steps were as follows: we extracted total RNA by TRIzol (Invitrogen, United States of America), and cDNA was synthesized using primers and PrimeScript RT Reagent Kit (TaKaRa, Japan). SYBR Green on a Light Cycler 480 Real-Time PCR System (Roche Diagnostics, Germany) was applied for qRT-PCR. Relative gene expression was calculated using the 2^-ΔΔCt^ method with GAPDH being the control for SERPINH1 and U6 for miR-29c-3p. The primer sequences applied are displayed in Table 1.

**Table 1:** Primer sequences in quantitative real-time polymerase chain reaction.

Gene	Sequence	Sequence
miR-29c-3p	Forward Primer	5’-TAGCACCATTTGAAATCGGTT-3’
	Reverse Primer	5’-GTGCAGGGTCCGAGGT-3’
U6	Forward Primer	5’-CTCGCTTCGGCAGCAC-3’
	Reverse Primer	5’-AACGCTTCACGAATTTGCG-3’
SERPINH1	Forward Primer	5’-TGCTAGTCAACGCCATGTTCT-3’
	Reverse Primer	5’-ATAGGACCGAGTCACCATGAA-3’
GAPDH	Forward Primer	5’-GTCAAGGCTGAGAACGGGAA-3’
	Reverse Primer	5’-AAATGAGCCCCAGCCTTCTC-3’

Source: Elaborated by the authors.

### Western blot assay

Steps specific to Western blot were as previously described^16^. Total proteins of cells were conducted, and the protein concentration was quantified using a BCA kit (Sigma-Aldrich, United States of America). Firstly, 20 μg proteins were separated by SDS-PAGE electrophoresis, then transferred to the PVDF membrane, and blocked with 5% skim milk for 1 hour. Primary antibodies presented in the assay were: rabbit anti-SERPINH1 (ab109117), rabbit anti-VEGFA (ab214424), rabbit anti-Wnt-1 (ab15251), rabbit anti-β-catenin (ab32572), rabbit anti-β-catenin (phospho S37) (ab75777), rabbit anti-AAK1 (ab134971), and rabbit anti-GAPDH (ab181602).

The following day, the membrane was rinsed with TBST buffer three times, and then incubated with the secondary antibody goat anti-rabbit IgG (ab6721) at room temperature for 2 hours. All were purchased from Abcam (United Kingdom). Finally, the signal was indicated by enhanced chemiluminescence (Beyotime, China), and relative concentration was evaluated by Quantity One (Bio-Rad, United States of America).

### Enzyme-linked immunosorbent assay for VEGFA concentration detection

The after-centrifugation supernatant was collected from each treatment group to detect VEGFA secretion in ESCC cells. The concentration of VEGFA was analyzed via an enzyme-linked immunosorbent assay (ELISA) kit complying with the manufacturer’s instructions (Proteintech Group, United States of America). The relative level of secreted VEGFA protein was normalized to the control level.

### Dual-luciferase reporter assay

A specific fragment containing the putative miR-29c-3p binding site on the 3’UTR of SERPINH1 mRNA (SERPINH1-Wt) was inserted into pmir-GLO (Promega, United States of America). SERPINH1-Wt (or SERPINH1-Mut) and miR-29c-3p mimic (or the negative control) were co-transfected with KYSE-150 cells. Complying with the instruction, we applied Lipofectamine 2000 (Invitrogen, United States of America) to transfect plasmids. Luciferase activity was evaluated through the Dual-Luciferase Reporter Assay System kit (K801-200, BioVision, United States of America) complying with the instructions provided by the manufacturer (Promega, United States of America).

### Flow cytometry

The transfected cells were resuspended and treated as single cells, and a control group was set up. One group was treated with Annexin V-FITC, and the other group was treated with PI staining solution. Subsequently, the cells in the experimental groups were added with Annexin V-FITC and PI staining solution, and finally detected on flow cytometry.

### Statistical analysis

Inter-group differences were evaluated via t-test or one-way analysis of variance on GraphPad Prism 6 software (GraphPad Software, United States of America). Pearson’s correlation analysis revealed the correlation between miR-29c-3p and SERPINH1. We repeated all experiments independently for three times. Statistical significance was suggested by *p* < 0.05. Data were presented in the form of mean ± standard deviation.

## Results

### miR-29c-3p is lowly expressed in ESCC tissues and cells

**Figure 1:**
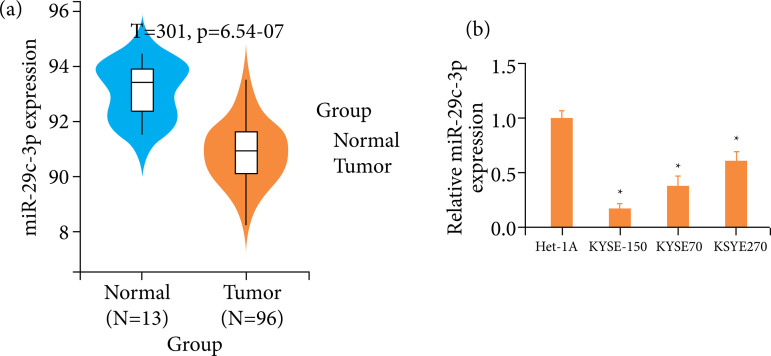
miR-29c-3p expression was relatively low in ESCC. **(a)** miR-29c-3p level in normal (blue) and ESCC tumor (yellow) samples. **(b)** miR-29c-3p mRNA level in human normal esophageal epithelial cells Het-1A and ESCC cell lines (KYSE-150, KYSE70, and KYSE270).

In various cancers, miR-29c-3p is identified to be dysregulated and to manipulate cancer progression^7^^,^ 8 ^,^ 17. The way miR-29c-3p impacted ESCC was explored via analyzing miR-29c-3p level in ESCC and normal tissue via bioinformatics means. In ESCC tumor tissues, miR-29c-3p mRNA level was illustrated to be remarkably lower than the normal tissues (Fig. 1a). Additionally, miR-29c-3p mRNA level in Het-1A and three ESCC cell lines were examined via qRT-PCR. It was revealed that cancer cell lines KYSE-150, KYSE70, and KYSE270 expressed lower miR-29c-3p level compared with Het-1A, among which miR-29c-3p expression was relatively low in KYSE-150, but high in KYSE270 (Fig. 1b). Thus, we chose these two cell lines for subsequent experiments. The already mentioned results implied a notable low expression of miR-29c-3p in ESCC.

ESCC: esophageal squamous cell carcinoma; *p < 0.05; ****p < 0.0001. Source: Elaborated by the authors.

### Aberrantly expressed miR-29c-3p affects the malignant progression of ESCC cells

**Figure 2:**
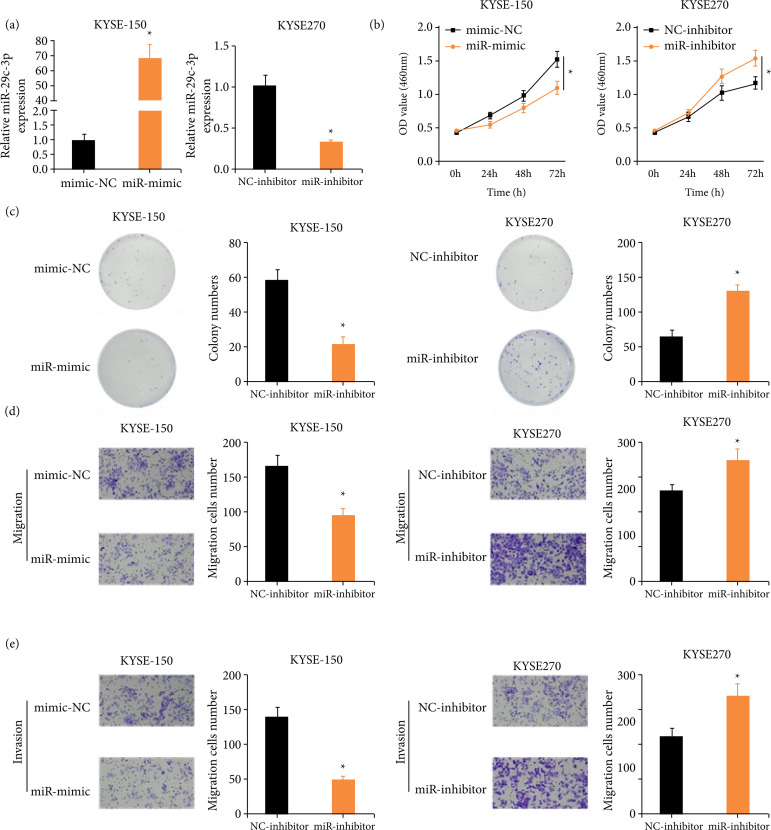
Aberrantly expressed miR-29c-3p affects proliferation, migration, and invasion of ESCC cells. **(a)** KYSE-150 and KYSE270 cells were transfected with mimic-NC/miR-mimic and NC-inhibitor/miR-inhibitor, respectively, with miR-29c-3p level detected in both groups. **(b)** The impact of miR-29c-3p on ESCC cell proliferation. **(c)** Colony formation ability of ESCC cells affected by abnormally expressed miR-29c-3p. (d and e) Dysregulated miR-29c-3p affected the migration and invasion of ESCC cells.

ESCC: esophageal squamous cell carcinoma; NC: negative control; OD: optical density; *p < 0.05. Source: Elaborated by the authors.

To explore the way aberrantly expressed miR-29c-3p affected ESCC cell proliferation, migration, and invasion, ESCC cell lines with overexpressed and silenced miR-29c-3p were constructed. miR-29c-3p mRNA level in ESCC cells was checked via qRT-PCR, which was markedly increased in the miR-mimic treatment group, but notably decreased in the miR-inhibitor treatment group (Fig. 2a). It was revealed that upregulated miR-29c-3p markedly repressed ESCC cell proliferation over the negative control (NC) group by applying CCK-8 and colony formation assays, while the opposite result was obtained with lowly-expressed miR-29c-3p (Figs. 2b and 2c). Thereafter, transwell assays uncovered that compared with the NC group, overexpression miR-29c-3p remarkably reduced cell migration and invasion, while knockdown miR-29c-3p yielded the opposite result (Figs. 2d and 2e). The results of flow cytometry showed that, compared with the mimic-NC group, the cell cycle of the miR-mimic group stagnated in the G0/G1 phase, and the ability of apoptosis was significantly increased. Compared with the NC-inhibitor group, the miR-inhibitor group exhibited cell cycle arrest in the S phase and significantly decreased apoptotic ability^18^. Jointly, miR-29c-3p notably suppressed ESCC cell malignant progression.

### Aberrantly expressed miR-29c-3p affects the angiogenesis of ESCC cells

**Figure 3:**
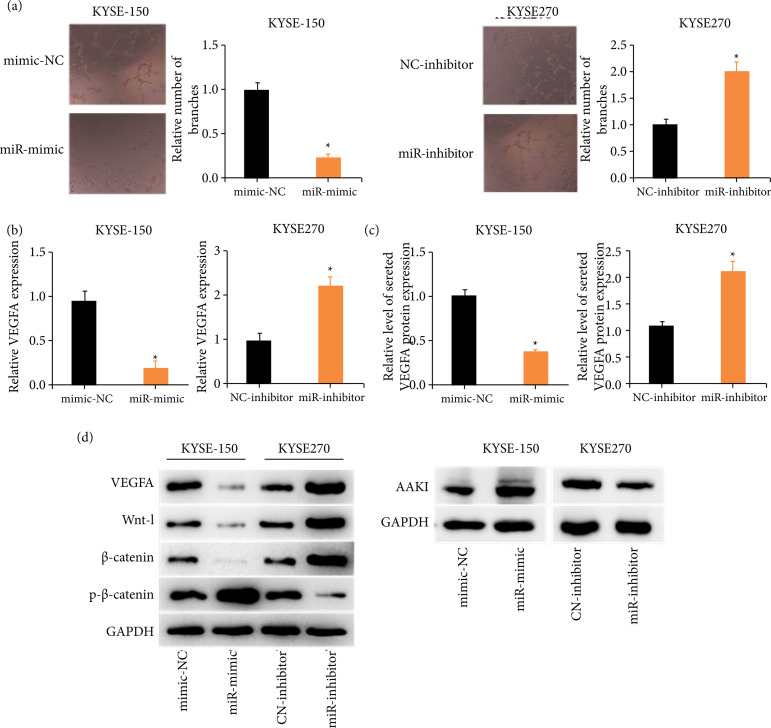
Aberrantly expressed miR-29c-3p affects the angiogenesis of ESCC cells. **(a)** HUVEC angiogenesis in groups treated with different ESCC-CM. **(b)** Angiogenesis marker gene VEGFA mRNA level in ESCC cells. **(c)** VEGFA protein level in ESCC cells were detected via enzyme-linked immunosorbent assay. **(d)** VEGFA and Wnt pathway-related proteins levels in ESCC cells were detected via Western blot.

NC: negative control; VEGFA: vascular endothelial growth factor A; ESCC: esophageal squamous cell carcinoma; CM: conditioned medium; *p < 0.05. Source: Elaborated by the authors.

The way aberrantly expressed miR-29c-3p influenced *in-vitro* angiogenesis of ESCC cells was revealed through treating HUVEC with CM containing overexpressed or silenced miR-29c-3p. The assay exhibited that ESCC conditioned medium CM with overexpressed miR-29c-3p (miR-mimic) remarkably repressed angiogenesis in comparison with the control CM (mimic-NC). However, the CM with silenced miR-29c-3p (miR-inhibitor) remarkably stimulated angiogenesis (Fig. 3a). Thereafter, VEGFA level in ESCC cells from different treatment groups was checked via qRT-PCR. VEGFA expression was remarkably lower upon up-regulating miR-29c-3p, while the opposite result was obtained upon silencing miR-29c-3p (Fig. 3b). Subsequently, VEGFA level in different conditioned media was individually examined via ELISA, whose results were consistent with qRT-PCR (Fig. 3c). Wnt signaling is one of the important pathways in the cellular process, so Wnt signaling pathway-related proteins and VEGFA protein expressions in ESCC cells were evaluated via Western blot. It was displayed that VEGFA, Wnt-1, and β-catenin expression levels in ESCC cells significantly were reduced, and the phosphorylation of β-catenin and AAK1 were stimulated upon upregulating miR-29c-3p, while silencing miR-29c-3p yielded the reverse results (Fig. 3d). To sum up, miR-29c-3p repressed angiogenesis and Wnt signaling pathway transduction in ESCC.

### miR-29c-3p represses ESCC cell proliferation, migration, invasion and angiogenesis via the Wnt signaling pathway

**Figure 4:**
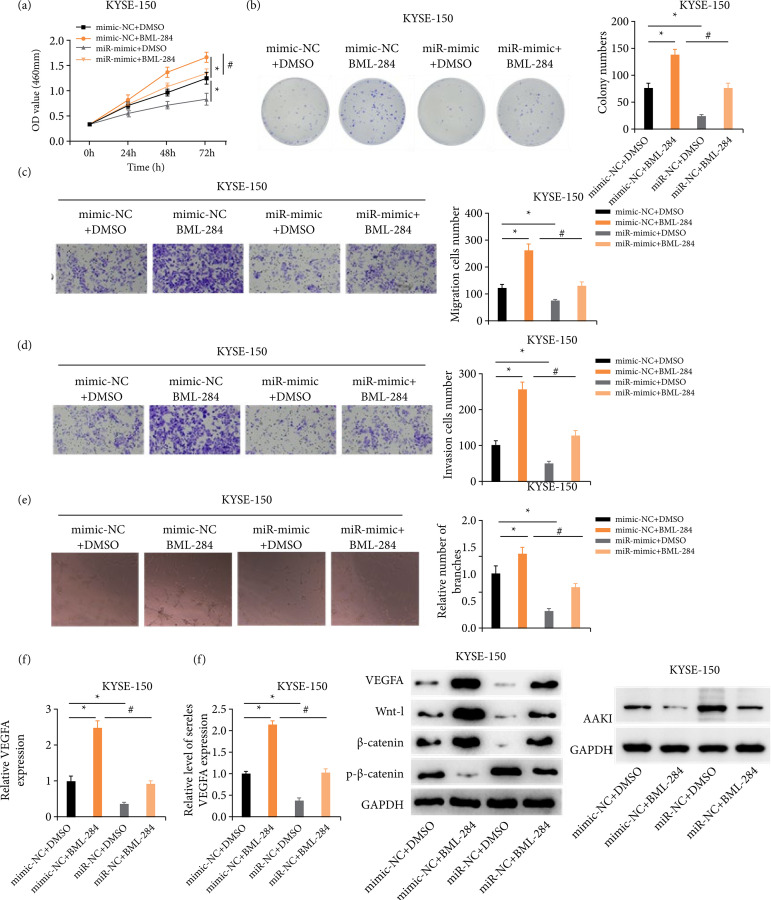
miR-29c-3p represses ESCC cell progression and angiogenesis through the Wnt pathway. **(a)** The effects of different treatments on ESCC cell proliferation. **(b)** Colony formation ability of ESCC cells with different treatments. (c and d) Effects of different treatments on migration and invasion of ESCC cells. **(e)** HUVEC angiogenesis in groups treated with different ESCC-CM. **(f)** Angiogenesis marker gene VEGFA level in ESCC cells. **(g)** VEGFA level in differently-treated ESCC cells were detected via enzyme-linked immunosorbent assay. **(h)** VEGFA and Wnt pathway-related protein levels in ESCC cells.

NC: negative control; OD: optical density; VEGFA: vascular endothelial growth factor A; ESCC: esophageal squamous cell carcinoma; CM: conditioned medium; *p < 0.05 *vs*. (mimic-NC+DMSO); #*p* < 0.05 *vs*. (miR-NC+BML-284). Source: Elaborated by the authors.

The way miR-29c-3p affected the Wnt signaling pathway was investigated via treating ESCC cells with BML-284, a Wnt pathway activator. Cell grouping was as follows: mimic-NC+DMSO, miR-mimic+DMSO, mimic-NC+BML-284, and miR-mimic+BML-284. The results of biological cell experiments suggested that BML-284 treatment remarkably enhanced the ESCC cell progression. Meanwhile, BML-284 treatment could reverse the repression of ESCC cell malignant behaviors caused by the up-regulated miR-29c-3p (Figs. 4a–4d).

The results of flow cytometry showed that, compared with the NC group, the cell cycle of the BML-284 group stagnated in the S phase, and that the miR-mimic group was staged in the G0/G1 phase. The level of the cell cycle after co-treatment of BML-284 and miR-mimic was similar to that of the NC group. Meanwhile, BML-284 repressed the ability of apoptosis was repressed by BML-284 and promoted by miR-mimic, while it was restored to the level of NC group after co-treatment of BML-284 and miR-mimic^18^. The angiogenesis assay manifested that BML-284 treatment markedly enhanced the angiogenesis ability of HUVEC, and BML-284 treatment could reverse the repression caused by the upregulated miR-29c-3p on angiogenesis (Fig. 4e). Moreover, BML-284 treatment could reverse the overexpressed miR-29c-3p-caused down-regulation of VEGFA in ESCC cells (Figs. 4f and 4g).

Detection via Western blot revealed VEGFA and Wnt pathway-related protein expression levels in ESCC cells. While VEGFA, Wnt-1, and β-catenin were remarkably upregulated, the p-β-catenin and AAK1 were downregulated since BML-284 treatment, and, after the introduction of overexpressed miR-29c-3p, the opposite results were obtained (Fig. 4h). It was suggested that miR-29c-3p repressed ESCC cell progression, as well as HUVEC angiogenesis through deactivating the Wnt signaling pathway.

### miR-29c-3p targets SERPINH1 in ESCC cells

**Figure 5:**
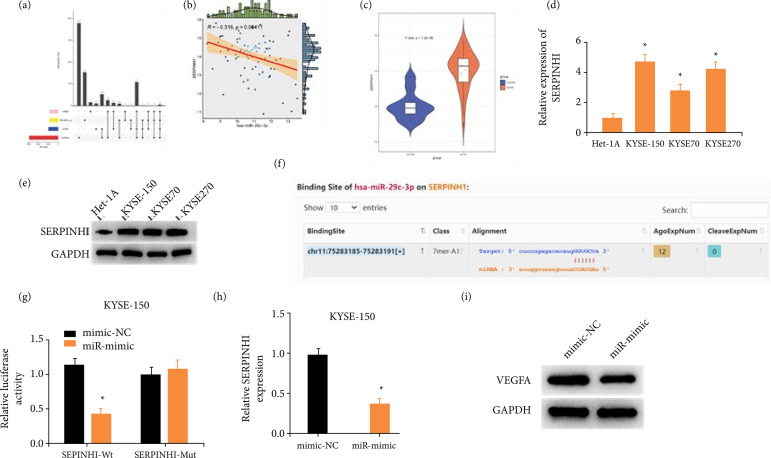
miR-29c-3p targets SERPINH1 in ESCC cells. **(a)** The miR-29c-3p downstream target genes were predicted by intersecting the differentially upregulated mRNAs with mRNAs in starBase, miRDB, and mirDIP databases. **(b)** Pearson’s correlation analysis results that miR-29c-3p and SERPINH1 were negatively correlated in ESCC tissues. **(c)** SERPINH1 levels in adjacent tissues (blue) and ESCC tumor samples (yellow). **(d)** mRNA and **(e)** protein levels of SERPINH1 in Het-1A and ESCC cell lines (KYSE-150, KYSE70, and KYSE270). **(f)** Bioinformatic analysis results indicated that miR-29c-3p could directly bind the 3’UTR of SERPINH1. **(g)** miR-29c-3p could directly bind the 3’UTR of SERPINH1 detected by dual-luciferase reporter assay. **(h)** mRNA and **(i)** protein expression levels of SERPINH1 were down-regulated after overexpressing miR-29c-3p.

NC: negative control; ESCC: esophageal squamous cell carcinoma; *p < 0.05; ****p < 0.0001. Source: Elaborated by the authors.

To further explore how miR-29c-3p regulated the Wnt signaling pathway in ESCC, through bioinformatics analysis, we predicted the target gene for miR-29c-3p. StarBase, miRDB, and mirDIP databases were intersected with differentially upregulated mRNAs, yielding that miR-29c-3p regulates the SERPINH1 gene (Fig. 5a). Pearson’s correlation analysis illustrated that SERPINH1 was notably negatively correlated with miR-29c-3p in ESCC (Fig. 5b). In ESCC tissues, SERPINH1 had a prominently higher expression than in adjacent normal tissues (Fig. 5c). *In-vitro* studies confirmed that SERPINH1 expression was elevated in ESCC cell lines over in Het-1A (Fig. 5d and 5e). A targeted binding site was predicted for miR-29c-3p and SERPINH1 (Fig. 5f). It was demonstrated that the luciferase activity of the SERPINH1-Wt reporter plasmid decreased by overexpressed miR-29c-3p through applying dual-luciferase reporter assay (Fig. 5g), while that of the mutant group did not change hugely, suggesting that miR-29c-3p targeted SERPINH1. qRT-PCR and Western blot suggested the mRNA and protein levels of SERPINH1 were prominently downregulated in the KYSE-150 cell line after treatment with miR-29c-3p mimic (Figs. 5h and 5i). These all indicated that SERPINH1 was upregulated in ESCC cells and a direct target of miR-29c-3p.

### SERPINH1 as a target gene of miR-29c-3p promotes KYSE-150 cell progression and angiogenesis

**Figure 6:**
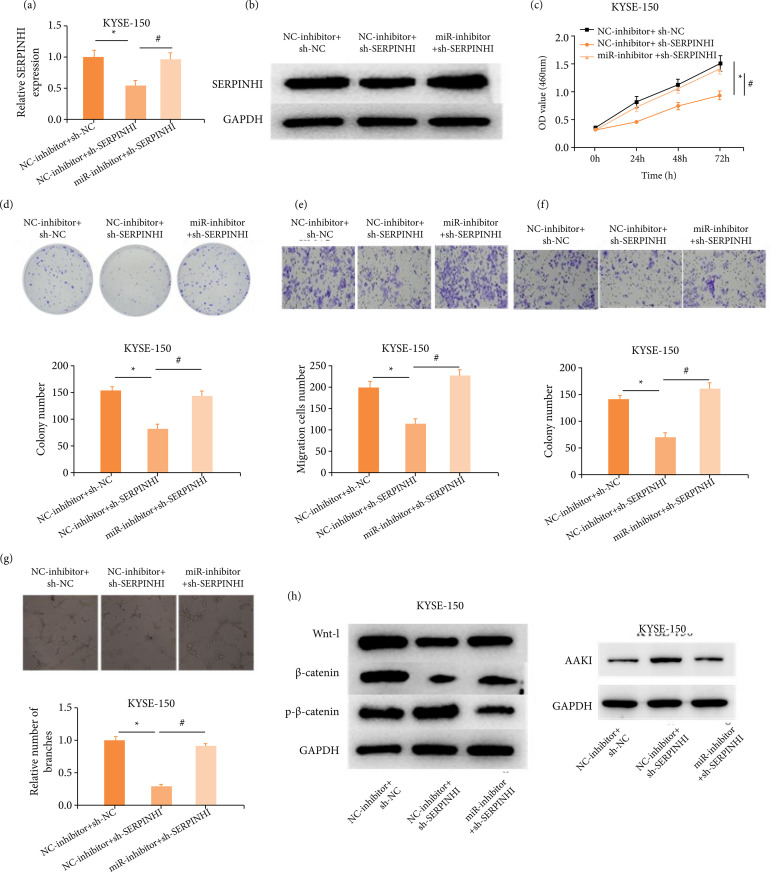
SERPINH1 as a target gene of miR-29c-3p promotes KYSE-150 cell progression and angiogenesis. KYSE-150 cells were transfected with sh-NC/sh-SERPINH1 and NC-inhibitor/miR-inhibitor. (**a and b**) The mRNA and protein levels of SERPINH1 were revealed by quantitative real-time polymerase chain reaction and Western blot. **(c)** The effects of different treatments on ESCC cell proliferation. **(d)** Colony formation ability of ESCC cells with different treatments. (**e and f**) Effects of different treatments on migration and invasion of ESCC cells. **(g)** HUVEC angiogenesis in groups treated with different ESCC-CM. **(h)** Wnt pathway-related protein levels in ESCC cells.

NC: negative control; OD: optical density; ESCC: esophageal squamous cell carcinoma; *p < 0.05 *vs*. (NC-inhibitor+sh-NC); #*p* < 0.05 *vs*. (NC-inhibitor+sh-SERPINH1). Source: Elaborated by the authors.

To verify that miR-29c-3p/SERPINH1 axis was associated with the development of ESCC, we treated KYSE-150 cells with sh-SERPINH1 and miR-inhibitor. As manifested in Figs. 6a and 6b, sh-SERPINH1 prominently repressed SERPINH1 mRNA and protein levels, which were then restored by miR-inhibitor treatment. CCK-8 (Fig. 6c) and cell colony formation (Fig. 6d) exhibited a notable repressed growth of KYSE-150 cells by the knockdown of SERPINH1, while simultaneous administration of miR-inhibitor could restore the growth of KYSE-150 cells. Similarly, the results of transwell (Fig. 6e and 6f) and angiogenesis assay (Fig. 6g) exhibited that the inhibition of migration, invasion, and angiogenesis abilities of KYSE-150 cells through sh-SERPINH1 was restored through miR-inhibitor treatment. These all suggested that SERPINH1 was a target gene for miR-29c-3p that promoted KYSE-150 cell progression and angiogenesis. The results of flow cytometry showed that, compared with the NC group, the cell cycle of the sh-SERPINH1 group stagnated in the G0/G1 phase, and the miR-inhibitor group can restore the level of the cell cycle to the NC group. Meanwhile, sh-SERPINH1 promoted the ability of apoptosis, but miR-inhibitor restored the ability of cell apoptosis to the NC group^18^.

To verify that miR-29c-3p/SERPINH1 axis regulated the Wnt signaling pathway in ESCC cells, we examined the expression of Wnt pathway-related proteins by Western blot, which manifested that inhibition with SERPINH1 remarkably suppressed levels of Wnt-1 and β-catenin protein, while increasing p-β-catenin and AAK1 protein levels over the NC-inhibitor+sh-NC group. Activation of the Wnt signaling pathway was restored through simultaneous inhibition of miR-29c-3p and SERPINH1 (Fig. 6h). Activation of the Wnt signaling pathway was repressed through sh-SERPINH1 in ESCC cells. It was demonstrated that miR-29c-3p/SERPINH1 axis was involved in regulating angiogenesis and malignant phenotype of ESCC cells through the Wnt signaling pathway.

## Discussion

At present, the major cause of poor clinical prognoses in ESCC patients is the lack of effective treatment. Hence, it is essential that the molecular mechanism of tumor progression be explored and unique promising therapeutic targets for ESCC be developed. Over the years, studies have demonstrated that miR-29c-3p plays anti-tumor effects on multiple cancers, but few reports revealed its functions in regulating ESCC malignant progression.

Our investigation into the manipulation of miR-29c-3p on tumor progression and angiogenesis in ESCC was presented herein. We revealed markedly low miR-29c-3p expression in ESCC tissues and cells, while upregulated miR-29c-3p remarkably reduced ESCC cell progression. Furthermore, notably low miR-29c-3p expression in bladder cancer was exhibited by Yu et al.^19^, which impedes cell migration and invasion. Lv et al.^20^ manifested that overexpressed miR-29c-3p remarkably constrained HCC cell migration and proliferation and might be a potential therapeutic target in HCC precise treatment. Those studies are consonant with this study, indicating that miR-29c-3p mainly suppressed cancer progression.

Angiogenesis is one of the important ways to boost underway tumor growth and metastasis^21^^,^ 22. Tumor angiogenesis largely depends on VEGFA-driven responses, which largely contribute to vasculature dysfunction^23^. Clinical observations demonstrated that VEGFA widely overexpresses in various tumor types, resulting in increased neovascularization and poor prognosis^24^. Therefore, the repressed VEGFA expression is often delivered as a therapeutic target for cancer angiogenesis. For instance, ACE2 represses breast cancer cell angiogenesis via downregulating VEGFA, as well as decreasing the phosphorylation of ERK1/2, MEK1/2, and VEGFR2 in HUVEC^25^. B7-H3 knockout could downregulate VEGFA to inhibit colorectal cancer angiogenesis^26^. In addition, Yu et al.^27^ exhibited that overexpressed PCDH8 repressed VEGFA secretion and consequently repressed angiogenesis in ESCC cells. Our study further confirmed that down-regulated VEGFA could repress angiogenesis, and miR-29c-3p constrained VEGFA level. This is consistent with the findings of Liu et al.^21^, that miR-29c (precursor of miR-29c-3p) can directly down-regulate VEGFA and suppress angiogenesis in lung adenocarcinoma cells.

Tumor angiogenesis is not only regulated by miRNAs, but also by bunches of signaling pathways including MAPK and Hedgehog^28^^,^ 29. As reported by others, the Wnt/β-catenin signaling pathway functions crucially on angiogenesis, while activating this pathway facilitates vascular cell proliferation and levels of angiogenic factors, including VEGF^30^^,^ 31. Accordingly, blocking this pathway is pivotal to the repression of tumor angiogenesis.

Our work revealed that miR-29c-3p repressed the Wnt signaling pathway in ESCC cells, which in turn reduced VEGFA secretion from tumor cells and suppressed angiogenesis in ESCC cells. The rescue assay further implied that BML-284, a Wnt pathway activator, reversed the repression of angiogenesis in ESCC cells caused by overexpressed miR-29c-3p. Moreover, other studies indicated that the Wnt pathway can be repressed to constrain tumor angiogenesis. For instance, miR-361-3p represses gastric cancer cell proliferation, invasion, and migration, and HUVEC angiogenesis via down-regulating HMGA1 to disrupt the Wnt/β-catenin pathway^32^. miR-1301 controlled the Wnt/β-catenin signaling pathway via BCL9, which reversely represses the migration, invasion, and angiogenesis of hepatocellular carcinoma^33^. These are consistent with our results, implying that repression of the Wnt pathway could constrain tumor angiogenesis.

Dysregulation of miRNAs is known to interfere with oncogenic or anti-cancer target gene expression, and involve in the pathogenesis of cancer^34^. To further investigate how miR-29c-3p manipulated the Wnt signaling pathway in ESCC, we discovered that miR-29c-3p targeted SERPINH1 and repressed SERPINH1 expression in ESCC cells via bioinformatics analysis and dual-luciferase reporter assay. In human pan-cancer, SERPINH1 has been found to be a crucial prognostic biomarker that correlates to tumor immunity^35^. We exhibited for the first time that SERPINH1 was involved in modulating ESCC cell biological function, and repression of SERPINH1 could abate the angiogenesis ability of ESCC cells. Consistent with our study, SERPINH1 was proven to facilitate angiogenesis in gliomas^36^. Our study also found that knockdown of SERPINH1 repressed Wnt signaling pathway activation, and this suppression was reversed with miR-29c-3p inhibitors. To conclude, miR-29c-3p repressed Wnt signaling pathway activation by SERPINH1 downregulation.

## Conclusion

To conclude, our study first demonstrated that SERPINH1 acted as an oncogene that controlled malignant phenotypes of ESCC cells, and the mechanism was elucidated at cellular level that miR-29c-3p repressed angiogenesis in ESCC targeting SERPINH1 to inactivate the Wnt signaling pathway. This not only deepened our understanding of ESCC angiogenesis, but also provided potential therapeutic targets for the precise treatment of ESCC. However, the lack of animal validation is a shortcoming of this study. In addition, more studies are needed to explore upstream target genes that regulate aberrant miR-29c-3p expression, thereby refining the regulatory network of miR-29c-3p in repressing tumor progression.

## Data Availability

All data sets were generated and analyzed in the current study. Supplemental figure is available at figshare. https:doi.org/10.6084/m9.figshare.24525868
