# Supplementary figures and images for: miR-29c-3p represses the angiogenesis of esophageal squamous cell carcinoma by targeting SERPINH1 to regulate the Wnt signaling pathway
Source: Acta Cir Bras. 2023 Dec 4;38:e385223. doi: 10.1590/acb385223 (PMC10695189; doi:10.1590/acb385223)

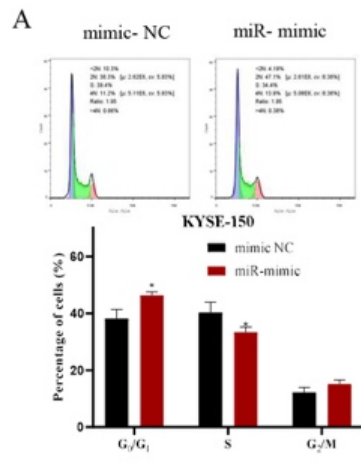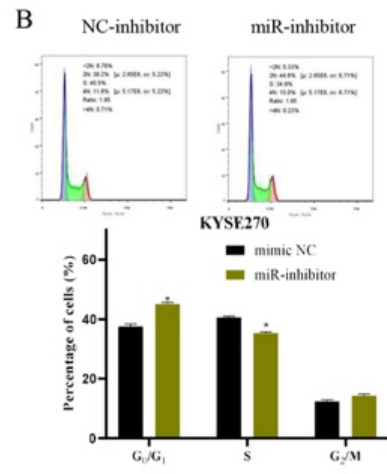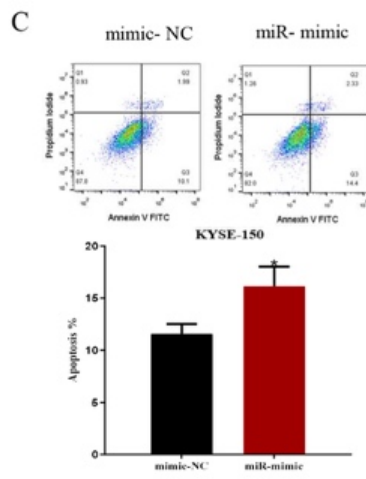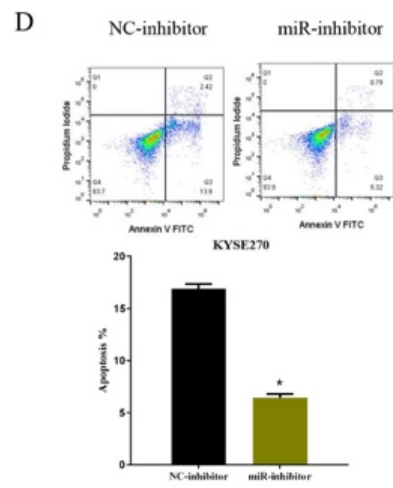

# KYSE-150

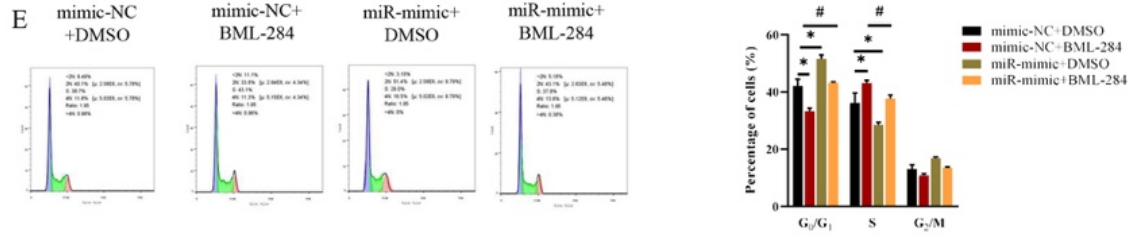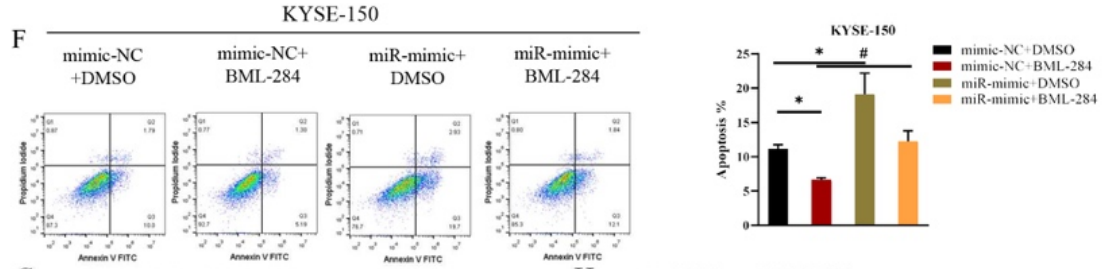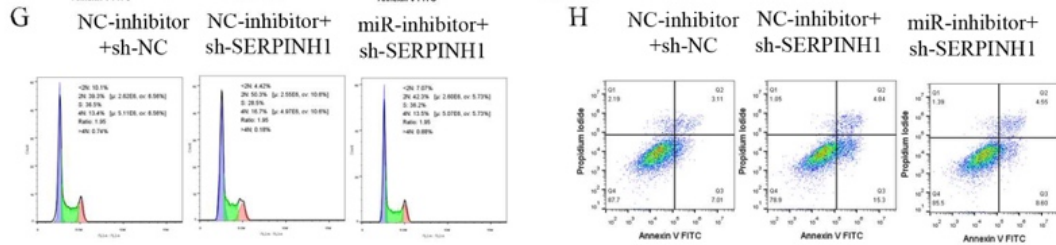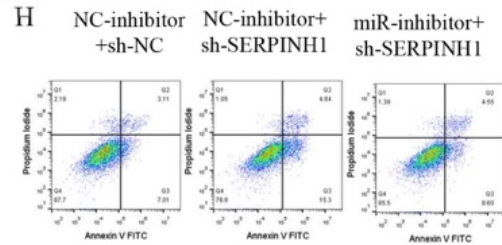

Supplement: Supplementary file 1 [file 1678-2674-acb-38-e385223-suppl1.pdf]
